# Study on radial fracture mechanism of saturated granite subjected to coupled static–cyclic impact loading: An experimental investigation

**DOI:** 10.1371/journal.pone.0340870

**Published:** 2026-01-15

**Authors:** Yunmin Wang, Xin Liu, Zhenyang Xu, HongLiang Tang

**Affiliations:** 1 State Key Laboratory of Safety and Health for Metal Mines, Sinosteel Maanshan General Institute of Mining Research Co., Ltd, Maanshan, Anhui, China; 2 Key Laboratory of Ministry of Education on Safe Mining of Deep Metal Mines, Northeastern University, Shenyang, Liaoning, China; 3 School of Mining Engineering, University of Science and Technology Liaoning, Anshan, Liaoning, China; 4 Engineering and Technology Research Center of High-Efficiency Mining-Processing and Utilization of Metal Mineral Resources, Anshan, Liaoning, China; 5 China safety technology research academy of ordnance industry, Beijing, China; Henan Polytechnic University, CHINA

## Abstract

The rock failure mode under coupled static-cyclic impact loading is not unified, and the radial fracture mode is a distinct type. In this study, cyclic impact tests were performed using a modified Split-Hopkinson Pressure Bar system under various axial pressures and confining pressures. Results showed that granite primarily exhibited radial tensile-dominated failure under coupled static-cyclic impact loading, with compression-shear failure at the specimen ends. Stress wave analysis revealed nonlinear propagation attributed to tensile stress waves. Numerical simulations confirmed that the velocity field exhibited a symmetrical transition with velocity vectors oriented in opposite directions on either side of a central zone. Two zones appear in the middle of the specimen in the displacement field, which correspond to the plastic stage in the stress-strain curve. Meanwhile, the free water in crack tips assists in enhancing crack propagation. In addition, before the final impact loading, the peak of the transverse relaxation time spectrum of micro-pores has returned to its initial state approximately. Fracture surface shows smoother surface under higher axial and confining pressures. The study provides reference for stability evaluation in underground engineering.

## Introduction

**Fig 1:**
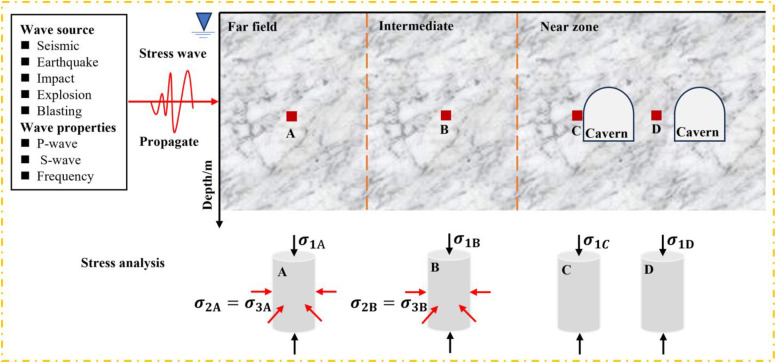
Overview of rock dynamics problems and influencing factors in underground engineering design [2].

A thorough understanding of the failure mechanism of rock under coupled static–cyclic impact loading is of great significance to the safety design and disaster prevention of underground rock engineering [1]. Typical rock dynamics issues related to the construction and utilization of an underground cavern are illustrated (Fig 1). Rocks are confined to different in situ stress environments at different locations from the tunnel, and the dynamic sources on confined rocks come from earthquakes, explosions, and impacts [2]. Due to the high cost of field tests, the Split-Hopkinson Pressure Bar (SHPB) based on experiment and numerical simulation is usually used to analyze the mechanical properties and failure characteristics of rock under coupled static–cyclic impact loading [3–4].

In the dynamic uniaxial compression test, the rock sample deforms along the radial direction, resulting in tensile splitting failure at low strain rates, characterized by the formation of new cracks emanating from the tips of preexisting defects. These cracks propagate in the direction of compressive stress, ultimately fusing. At high strain rates, rock specimen presents a crushing destruction mode and the fragment size decreases with the increase in strain rate [4–5]. In the triaxial compression SHPB tests, many factors affect the failure characteristics of rock, among which the most important factors are precompression axial pressure, confining pressure, strain rate, and frequent impact disturbances [6–11]. At low confining pressure or high strain rate, the rock also exhibits a predominant tendency toward tensile failure, accompanied by the fragmentation of the rock into relatively fine particles. At high confining pressure, the angle between the crack extension direction and the loading direction increases with the confining pressure, and the failure mode of the rock sample undergoes a transition from tensile-dominated to shear-dominated. Because the confining pressure can weaken the dilation of the rock sample, the normal stress of the rock sample increases, causing the frictional bearing capacity of cracks to exceed the cohesive stress of the rock sample. Therefore, the confining pressure has been shown to inhibit crack extension, while the strain rate has been demonstrated to promote fragmentation [4,5,12–14]. ‌Especially, the rock sample tends to compression-shear failure when the axial pressure is greater than the confining pressure [15]. However, under cyclic impact loading, the rock failure mode presents various characteristics (Table 1). In the majority of cases, the failure modes of shear failure and compression-shear failure are indispensable.

**Table 1:** Dynamic failure mode of rock subjected to confinement.

References	Rock (site)	Axial pressure (MPa)	Confining pressure (MPa)	Failure mode	Loading method
Gong et al. 2011 [15]	Sandstone (-)	60, 80, 100	5, 10	compression-shear failure	S
Liu et al. 2014 [5]	Amphibolite (Qinling)	–	2, 4, 6	shear failure	S
Hokka et al. 2016 [12]	Gray granite (Kuru)	5, 10, 20, 30, 50, 75	5, 10, 20, 30, 50, 75	shear failure	S
Gong et al. 2019 [4]	Sandstone (Linyi)	5, 7.5, 10, 12.5, 15	5, 7.5, 10, 12.5, 15	shear failure	S
Yuan et al. 2020 [13]	Sandstone (-)	–	0.5, 2, 5, 10, 15, 20, 25	shear failure	S
Du et al. 2020 [14]	Sandstone (Neijiang)	7, 14, 21, 28	7, 14, 21, 28	tensile-dominated failure to the shear-dominated failure	S
Kong et al. 2020 [16]	Coal (Huaibei)	1.5, 3, 5, 7, 9	5	Tensile failure	S
Wang et al. 2019 [6]	Serpentine (Tongling)	100, 120, 140, 160	15, 20, 25, 30	tensile failure, shear failure, and pull-compression mixed friction failure	C
Wang et al. 2021 [7]	Sandstone (-)	–	5, 10	compression-shear failure	C
Ma et al. 2022 [8]	combined coal-rock bodies (-)	5	5, 10, 15	shear failure	C
Wang et al. 2023 [9]	Granite (Qinling)	2, 4, 6	4	Tensile failure	C
Zhang et al. 2024 [10]	Limestone (-)	6, 12, 24, 36, 48	5, 7.5, 10, 15	tensile failure to tensile-shear failure	C
Sun et al. 2025 [11]	Coal (Erdos)	18	23	tensile and compressive-shear composite failure	C

**Note:** (1) S: single impact loading; (2) C: cyclic impact loading; (3) Kong et al. 2020: the samples were filled with gas (CH_4_) under gas pressure (0.25 MPa, 0.50 MPa, 0.75 MPa, 1.00 MPa, 1.25 MPa, and 1.50 MPa)

**Fig 2:**
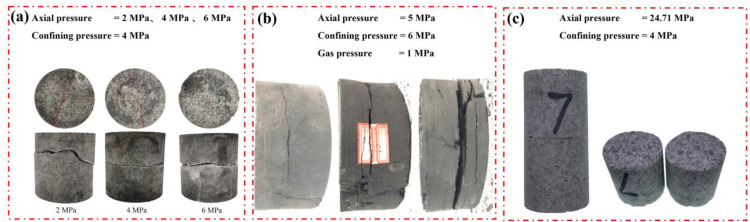
The axial tensile failure in the triaxial compression SHPB tests. (a) Wang et al. [9]. (b) Kong et al. [16]. (c) Our tests.

Unexpectedly, the radial fracture mode is observed by Wang et al. [9] (Fig 2a) and Kong et al. [16] (Fig 2b) in the triaxial compression SHPB tests, and such a phenomenon is also observed in our experiment (Fig 2c). Wang et al. [9] and Kong et al. [16] judged that axial tensile failure is primarily caused by the tensile stress wave reflected from the incident stress wave. However, it is imperative to note that the wave impedance of the incident bar and the specimen is not equivalent, which invariably gives rise to the occurrence of reflected waves. At present, there is a lack of detailed analysis regarding the radial fracture mechanism in coupled static–cyclic impact loading.

Therefore, a rationale is necessary to explain the absence of similar findings in other experiments. In this study, the radial fracture mechanism of saturated granite under coupled static–cyclic impact loading through experimental tests, stress wave analysis, numerical simulations, and microscopic characterization is systematically investigated.

## Experiments

### Sample preparation

**Fig 3:**
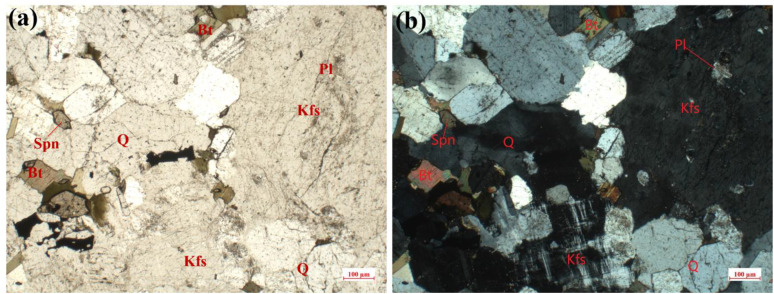
Thin section authentication and mineral composition of granite [17]. (a) Plane-polarized light. (b) Cross-polarized light.

The sesame white granite, commercially designated as G603, was extracted from a quarry in Rizhao. The granite is taxonomically classified as a microfine-grained black monzogranite, a designation that is contingent upon the meticulous geological classification criteria. The texture of the granite has been described as subhedral granular, with a clumpy structure. Its mineralogical composition has been identified as biotite, plagioclase, potassium feldspar, and quartz. The identification of these primary mineral constituents was conducted through the utilization of polarization microscopy (LEICA DM2500 p). The mineralogical composition is delineated (Table 2 and Fig 3), and detailed mechanical parameters of the tests are listed (Table 3) [17]. In addition, the samples were saturated for 8 hours through a vacuum water saturation device.

**Table 2:** Mineral characteristics of granite [17].

Minerals	proportion (%)	Mineral characteristics and descriptions
Quartz	54	Quartz is colorless and granular with grains of different shapes and sizes of up to several millimeters, and it has slight brittle breakage and ductile deformation.
K-feldspar	23	The K-feldspar mainly contained orthoclase and perthite shaped as hypidiomorphic and heteromorphic plate columnar with a particle size of 0.38–1.42 mm, and slight clayification generally occurs.
Plagioclase	15	Plagioclase is produced in the shape of automorphic and hypidiomorphic plate columnar with a particle size of 0.45–1.05 mm, and slight sericitization and clarification generally occur.
Biotite	5	The biotite had a characteristic interference color and was shaped like a yellowish-brown scale-like aggregate with a particle size of 0.04–0.21 mm, and extremely complete cleavage was developed.
Others	3	Small amounts of columnar zircon and automorphic titanite grains were visible, as were small amounts of stellate, agglomerate, and irregular opaque metallic minerals.

**Table 3:** Relevant granite static mechanical parameters.

Type	Density kg/m^3^	Elastic modulus GPa	Uniaxial compression strength MPa	Tensile strength MPa	Poisson’s ratio	P-wave velocity m/s
**Dry**	2643	42.7	158.3	6	0.256	4054
**Saturation**	–	19.6	150.6	–	0.166	–

### Test device

**Fig 4:**
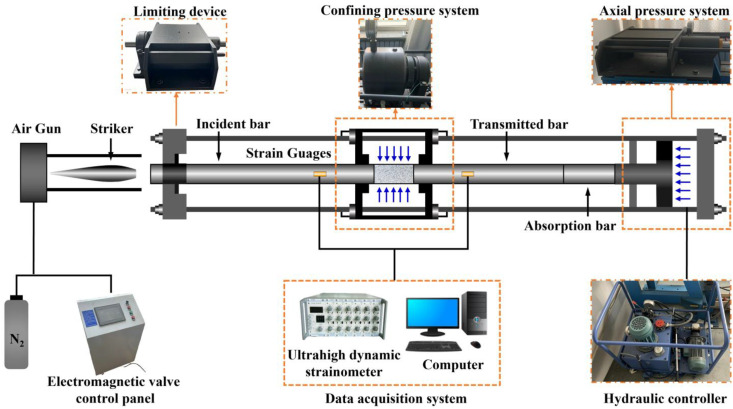
Schematic diagram of SHPB system.

The conventional SHPB experiment is modified by the incorporation of an axial limiting device for the bar and a high-pressure cylinder in the radial direction of the specimen (Fig 4). The hydraulic oil is introduced into the axial and radial cylinders through a hand-electric integrated pressurized pump, and the working pressure ranges from 0 to 63 MPa. The incident bar (50 mm in diameter) is 2.1 m in length, and the transmission bar is 1.8 m. Both bars are composed of low-alloy ultra-high-strength steel, and the modulus is 210 GPa and the density is 7900 kg/m³. The strain gauges employed for the monitoring of incident and transmitted waves are affixed in a radially symmetric position at the center of the bar. The voltage signals of the strain gauge on the incident and transmission bars are collected using an ultrahigh dynamic strain meter (LK2109A) and a high-speed data acquisition device (LK2400).

The initial step involves the installation of the specimen within the enclosure, ensuring its precise alignment with the central axis. Concurrently, the lateral of the system support components is adjusted, ensuring the lateral auxiliary longitudinal bars are firmly secured in their intended positions. The oil pressure pipelines should be connected in succession according to the system configuration. This will ensure that each of the shut-off valves is closed before each system is put into operation. Thirdly, before initiating the operation of the hand-operated pressure pump, it is essential to ensure that the hydraulic oil is introduced into the system. The quantity of oil introduced must exceed two-thirds of the oil tank’s total capacity. Fourthly, it is imperative to ensure the integrity of the electrical connection, with meticulous attention to grounding. The next step is to position the pressure control valve at the central point. The initiation of the loading process is contingent upon the implementation of a pressure-limiting valve. The initial step involves opening the upper closure of the pressure vessel. Once the activation button of the pressure pump station is depressed, the process of oil introduction will commence. It is imperative to closely monitor the capacity of the pressure vessel during this phase, ensuring that the upper closure of the pressure vessel is securely fastened to guarantee its integrity. Sixthly, the maximum pressure permitted for the electric pressure pump station is 12 MPa. If a pressure greater than 12 MPa is required, manual pressure augmentation is necessary. The maximum pressure that can be achieved through manual augmentation is less than 60 MPa. Seventhly, upon completion of the pressure augmentation, the bypass valve and the oil cylinder cutoff valve are to be closed, and the check valve of the pressure augmentation pump station is to be returned to the central position. Eighthly, the three-dimensional pressure augmentation shock experiment is initiated. Following the conclusion of the experiment, the pressure relief valve will begin to return to its original position, and the oil cylinder will begin to return to its original position. The oil pump will return the hydraulic oil through the oil pipes and the precise oil filter unit to the system oil tank. Once the hydraulic oil has been exhausted from the system and the pressure vessel, the experiment will be concluded, and the next experiment can be initiated.

### Test method

The cyclic impact compression tests were conducted on granite specimens by using the SHPB coupled static–dynamic loading system. The samples were tested under the hydraulic oil pressure of 1 MPa, 2 MPa, 3 MPa, 4 MPa, and 5 MPa. It is imperative to acknowledge that the magnitude of the confining pressure is equivalent to the value obtained from the oil pressure gauge located on the confining pressure device. Conversely, the axial pressure is determined by the value that is derived from the conversion of the reading from the oil pressure gauge on the axial pressure device [18], employing the established formula as follows,


$$P_{s}=5.168P_{O}+4.033$$ (1)

Where *P*_s_ is the axial pressure, *P*_o_ is the oil pressure, and the results after conversion are listed (Table 4).

**Table 4:** The experiment matches the actual parameters.

No.	Oil pressure (MPa)	Axial pressure (MPa)	Confining pressure (MPa)	*r*/*a*	Hydrostatic pressure (MPa)	Theoretical depth (m)
**Test 1**	1	9.20	1	1.1	4.13	156.20
**Test 2**	2	14.37	2	1.2	7.40	279.88
**Test 3**	3	19.54	3	1.2	10.7	404.69
**Test 4**	4	24.71	4	1.2	12.73	481.47
**Test 5**	5	29.87	5	1.2	15.39	582.07

**Fig 5:**
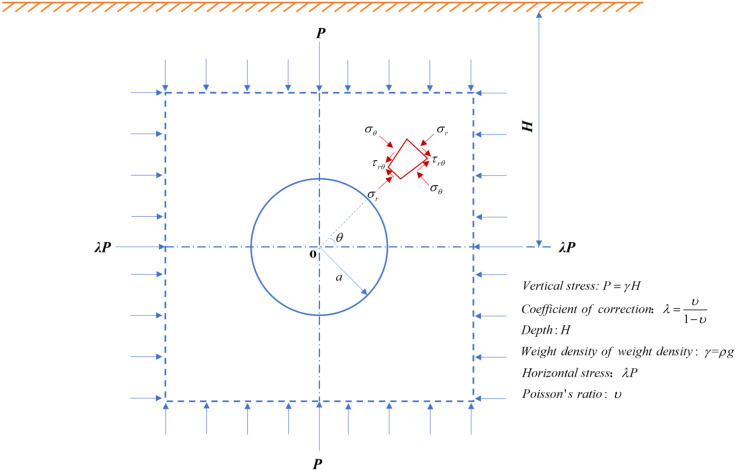
The stress distribution of the circular tunnel.


The axial pressure and confining pressure of the experimental setting should be consistent with the actual engineering so that the engineering research can have certain practical significance. A simple estimation can be made through theoretical methods. For example, a circular tunnel with a radius of *a* is excavated at a depth of *H* from the surface, where *H* is much larger than *a*. The problem can be considered a bidirectional, pressurized, and infinite plate orifice stress distribution problem (Fig 5). The element at *r*, with an angle *θ* to the horizontal axis, is taken from the center of the circular tunnel. The stresses at this location are as follows [19],

$$\begin{array}{c} \left\{ \;\;\;\begin{array}{c} \sigma_{r}=\frac{\text{1}}{\text{2}}\left(1+\lambda \right)P\left(1-\frac{a^{2}}{r^{2}}\right)+\frac{\text{1}}{\text{2}}\left(\lambda -1\right)P\left(1-4\frac{a^{2}}{r^{2}}+3\frac{a^{4}}{r^{4}}\right)\cos2\theta \\ \sigma_{\theta }=\frac{\text{1}}{\text{2}}\left(1+\lambda \right)P\left(1+\frac{a^{2}}{r^{2}}\right)-\frac{\text{1}}{\text{2}}\left(\lambda -1\right)P\left(1+3\frac{a^{4}}{r^{4}}\right)\cos2\theta \\ \tau_{r\theta }=\frac{\text{1}}{\text{2}}\left(1-\lambda \right)P\left(1+2\frac{a^{2}}{r^{2}}-3\frac{a^{4}}{r^{4}}\right)\sin2\theta \end{array}\right. \end{array}$$ (2)

Where *σ*_*r*_ is the axial stress, *σ*_*θ*_ is the circumferential stress, *P* is the initial vertical stress acting on the rock mass, and *λ* is the lateral pressure coefficient.

**Fig 6:**
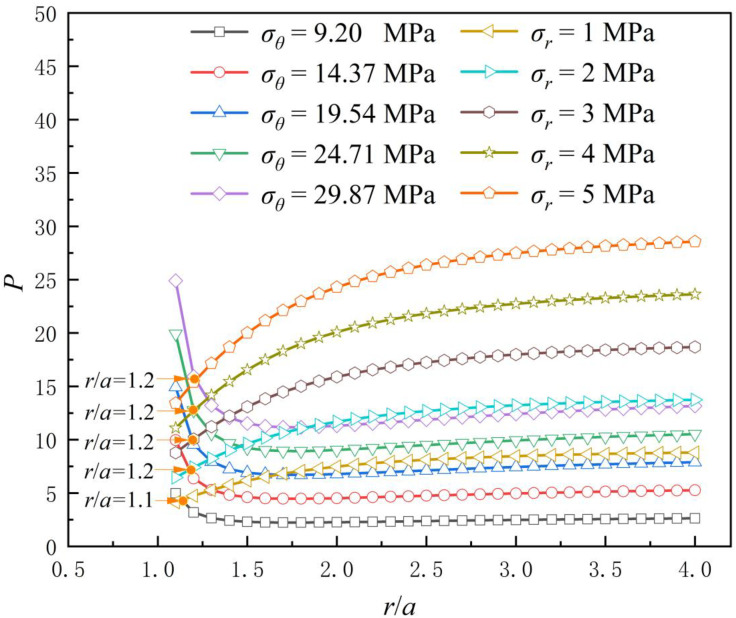
The relationship between *P* and *r/a.*

Due to the redistribution of stresses around the tunnel resulting from tunnel excavation, the axial and circumferential stresses differ at various locations around the tunnel. If the depth is constant, *p* is a fixed value, and *θ* is assumed to be 0 for ease of calculation. The axial stress due to stress redistribution in the tunnel is the confining pressure in the SHPB, and the circumferential stress is the axial pressure in the SHPB. Using the formula (2), the relationship between *P* and *r/a* can be obtained (Fig 6). It can be seen that the same *p* occurs when *r*/*a* is 1.1 and 1.2. The depths corresponding to the actual tunnel are 156.20 m, 279.88 m, 404.69 m, 481.47 m, and 582.07 (Table 4).

## Theoretical analysis and data processing of the SHPB coupled static–dynamic loading

### Theoretical analysis of the SHPB coupled static–dynamic loading

Li et al. [1] considered the influence of the pre-compression stress on the wave propagation in the SHPB based on one-dimensional wave theory. The equation of motion can be written as,


$$-\frac{\partial \left(P_{s}+P_{d}\right)}{\partial x}\Delta x=\rho A\Delta x\frac{\partial^{2}u}{\partial t^{2}}$$ (3)

where *A* is the area of the cross-section, *ρ* is the density of the material, *u* is the axial translational displacement, *P*_*s*_ is the axial static load, and *P*_*d*_ is the impact loading.

According to solid mechanics and wave theory,


$$\sigma =\frac{P_{s}+P_{d}}{A},\;\varepsilon =-\frac{\partial u}{\partial x},\;C=\sqrt{\frac{\text{1}}{\rho }\frac{d\sigma }{d\varepsilon }}$$ (4)

The axial static load is constant,


$$\frac{\partial P_{s}}{\partial x}=\frac{\partial P_{s}}{\partial t}=0$$ (5)

So,


$$\frac{\partial^{2}u}{\partial t^{2}}=C^{2}\frac{\partial^{2}u}{\partial x^{2}}$$ (6)

where *C* is the wave velocity in the pre-compression material.

**Fig 7:**
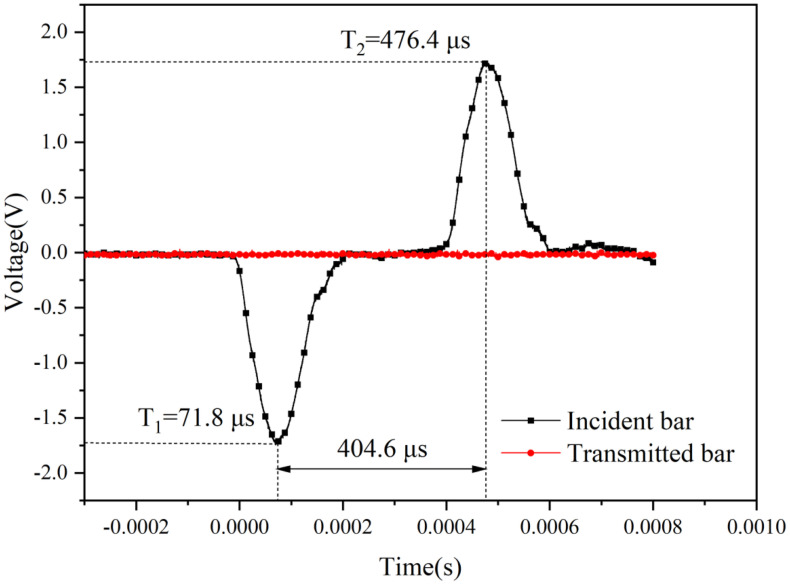
The methods for measuring wave velocity with the bar.

There are two methods for measuring wave velocity with the bar [20]. One method requires docking the incident bar with the transmission bar without placing the specimen. The wave velocity in the bar is obtained by dividing the distance between the strain gauges on the incident bar and the transmission bar by the time difference between the peaks of the incident wave and the transmitted wave. This method is also commonly used for calibrating the experimental system. The second method involves considering only the incident bar. The wave velocity in the bar is obtained by dividing twice the distance from the strain gauge of the incident bar to the free end face by the time difference between the peaks of the incident wave and the reflected wave. In this experiment, the length of the incident bar is 2.1 m, and two strain gauges are placed at a symmetric position halfway along its length to measure the incident and transmitted wave signals (Fig 7). Under the condition of non-axial pressure, the longitudinal wave velocity in the bar is 2.1 m/ 404.6 μs = 5190.3 m/s.

**Fig 8:**
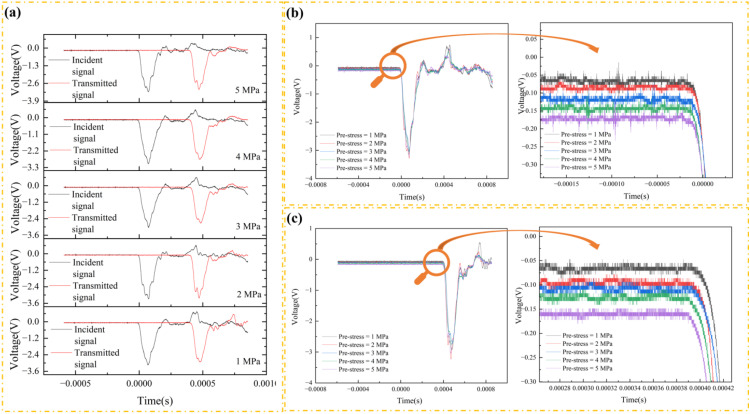
Stress wave with different axial oil pressure.

Assuming the axial pressure does not exceed the elastic limit of the material, then the material is in elastic compression, and the modulus of elasticity will generally remain constant. Axial pressure causes the material to compress axially and expand laterally, resulting in a slight decrease in total volume and an accompanying slight increase in density. According to formula (4), the wave velocity decreases under axial pressure. The length of the transmitted bar is 1.8 m, and the strain gauges are positioned in the middle. The distance from the strain gauges of the incident bar to the strain gauges of the transmitted bar is 1.95 m. The time difference between the peaks of the incident wave and the transmitted wave under different axial pressures is 402 μs, 404 μs, 404 μs, 404 μs, 403 μs, and 403 μs, and the computed average wave velocity is 4836.3 m/s (Fig 8).

**Fig 9:**
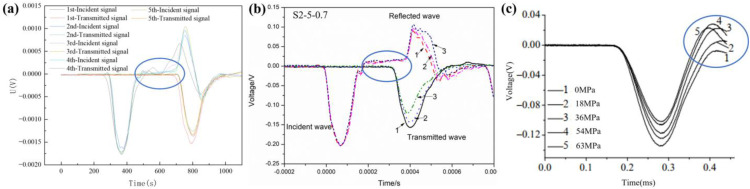
Effect of static axial pressure on the incident wave. (a) Test 5. (b) Wang et al. [7]. (c) Jin et al. [21].

The voltage jumping baseline of the incident and transmitted waves is affected by axial pressure without placing the specimen, and the value of the baseline increases with the axial pressure. After placing the specimen, the baseline of the voltage of the incident and transmitted waves is basically at 0 V. One possible reason is that the bar behaves like an elastic body when the specimen is not placed, and the bar is deformed by the axial pressure. When the specimen is placed, the bar becomes a rigid body, and the axial pressure mainly acts on the specimen. It is consistent with most experimentally observed waveforms (Fig 9). At the same time, a jump phenomenon occurs at the tail end of the incident wave, and the voltage baseline of the reflected wave is not 0 V.

Based on the first basic assumption of SHPB experiment technology (one-dimensional stress wave assumption) [20], the strain rate, strain, and stress can be calculated as follows,


$$\dot{\varepsilon }=\frac{C}{L_{s}}\left[\varepsilon_{i}\left(t\right)-\varepsilon_{r}\left(t\right)-\varepsilon_{t}\left(t\right)\right]$$ (7)

$$\varepsilon =\frac{C}{L_{s}}\int_{0}^{t}\left[\varepsilon_{i}\left(t\right)-\varepsilon_{r}\left(t\right)-\varepsilon_{t}\left(t\right)\right]$$ (8)

$$\sigma =\frac{A_{b}}{\text{2}A_{s}}E_{b}\left[\varepsilon_{i}\left(t\right)+\varepsilon_{r}\left(t\right)+\varepsilon_{t}\left(t\right)\right]$$ (9)

where *ε*_*i*_(*t*), *ε*_*r*_(*t*), *ε*_*r*_(*t*) are the incident, reflected, and transmitted strains, respectively. *E*_*b*_, *A*_*b*_, and *C* are the elastic modulus, cross-sectional area, and elastic wave velocity of the bars, respectively. *A*_*s*_, *L*_*s*_ are the cross-sectional area and height of the joint specimen, respectively. *t* is the duration of the elastic wave.

The second fundamental assumption based on the SHPB experimental technique (the assumption of uniform stress distribution of the specimen) [20], namely:


$$\varepsilon_{i}\left(t\right)+\varepsilon_{r}\left(t\right)=\varepsilon_{t}\left(t\right)$$ (10)

It is simplified as,


$$\dot{\varepsilon }=-\text{2}\frac{\text{C}}{{\text{L}}_{\text{s}}}\varepsilon_{r}(t),\;\varepsilon =-\text{2}\frac{\text{C}}{{\text{L}}_{\text{s}}}\int_{\text{0}}^{\text{t}}\varepsilon_{r}(t),\;\sigma =\frac{{\text{A}}_{b}}{{\text{A}}_{\text{s}}}E_{b}\varepsilon_{t}(t)\;$$ (11)

However, for the stress balance condition, Chen et al. [22] gave the judging criteria,


$$\sigma_{s}<\frac{\rho v_{bul}L_{bul}C}{2\left(L_{int}-L_{rp}\right)}\;or\;v_{bul}>\frac{2\left(L_{int}-L_{rp}\right)\sigma_{s}}{\rho L_{bul}C}\Rightarrow \sigma_{t}=\sigma_{i}+\sigma_{r}$$ (12)

$$\sigma_{s}>\frac{\rho v_{bul}L_{bul}C}{2\left(L_{int}-L_{rp}\right)}\;or\;v_{bul}<\frac{2\left(L_{int}-L_{rp}\right)\sigma_{s}}{\rho L_{bul}C}\Rightarrow \sigma_{t}=\sigma_{i}+\sigma_{r}-\sigma_{s}$$ (13)

Where *σ*_*s*_ is the axial pressure, *v*_*bul*_ is the striking velocity, *L*_*bul*_ = 0.34 m is the length of the striker, *L*_*int*_ = 2.1 m is the length of the incident bar, *L*_*rp*_ = 0.83 m is the distance between the reaction plate and the strain gauge. For example, *σ*_*s*_ = 29.87 MPa is chosen to calculate, then the threshold value of the striker velocity *v*_*bul*_ is 5.48 m/s.

In experimental configurations where the SHPB system remains constant, including the length of the striker bar, the characteristics of the incident wave are predominantly governed by the impact velocity. This is due to the principles of one-dimensional stress wave propagation theory [20], then,


$$\varepsilon_{i-\max}=\frac{v_{bul}}{2C}$$ (14)

From the formula (14) and the stress wave data, we can see that the striker velocity in the experiment is 16.61 m/s. This indicates that the original stress balance method can be adopted, as shown in formula (12).

### The data processing of the SHPB coupled static–dynamic loading

**Fig 10:**
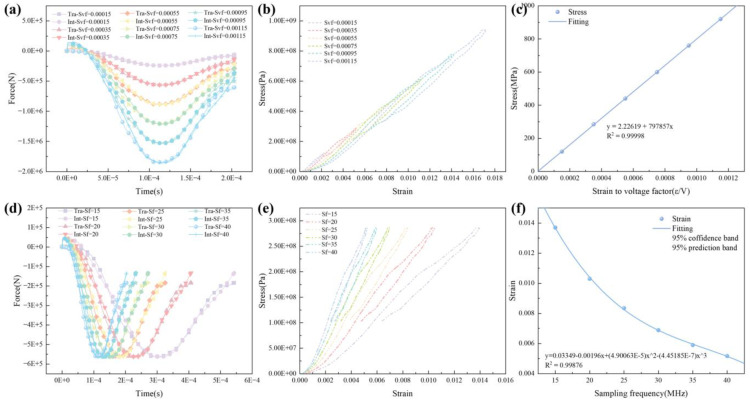
The influence of parameter selection on the results of data processing.

Due to the large amount of experimental data, processing it manually is time-consuming and labor-intensive. Additionally, there is a margin of error in determining the time alignment of the three waves while searching for the moment the wave starts and the moment of stress balance. Francis et al. [23] provided open-source software written in Matlab®, but two parameters must be set accurately; otherwise, it will affect the calculation of the stress-strain curve, specifically the strain-to-voltage factor (Svf) and sampling frequency (Sf). The parameter selection in the software is of crucial importance. The selection of Svf and Sf does not affect the stress balance conditions (Fig 10a and 10d), but has a certain influence on stress and strain. With the increase of Svf, both stress and strain increase (Fig 10b and 10c). With the increase of Sf, the stress remains unchanged and the strain decreases (Fig 10e and 10f).

**Fig 11:**
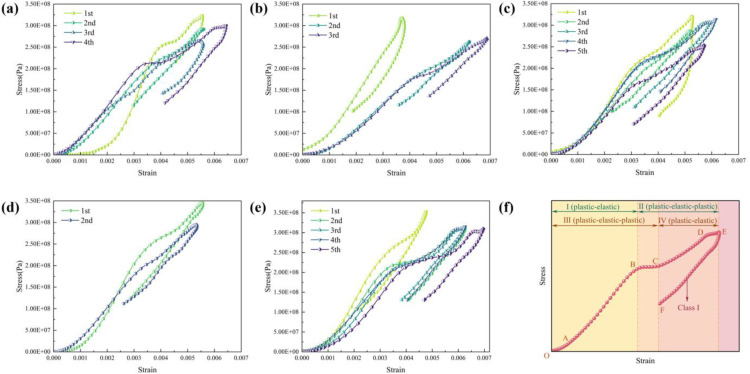
Cyclic impact dynamic stress-strain curves for specimens. (a) Test 1. (b) Test 2. (c) Test 3. (d) Test 4. (e) Test 5. (f) The general characteristics of the dynamic stress-strain curve.

The stress-strain curves can reveal the strength and deformation characteristics of rocks (Fig 11). The dynamic stress-strain curves of rocks under dynamic cyclic impacts have a typical weakening phenomenon with the increase of the number of impacts, which is consistent with the fit conclusion of most of the research. According to the dynamic stress-strain curve of rock can be divided into six stages (Fig 11f), OA stage is pore-fracture compaction, AB stage is elastic deformation, BC stage is unstable development of cracks, CD stage is experiencing elasticity stage again, DE stage is experiencing unstable development of cracks again, and EF stage is rebound. According to the morphology presented in different stages of the dynamic stress-strain curve of rocks, two types can be classified, i.e., the dynamic stress-strain curves are combined by plastic-elastic and plastic-elastic-plastic (Ⅰ-Ⅱ), or plastic-elastic-plastic and elastic-plastic (Ⅲ-Ⅳ).

## Energy evolution characteristics

According to the one-dimensional theory of stress waves and energy, the dissipated energy can pass through the compression bar system when the spatter kinetic energy of the rock specimen is ignored. The incident, reflected, and transmission energies are calculated from the following formula [20]:


$$W_{i}=\int_{0}^{t}A_{b}\sigma_{i}(t)C\varepsilon_{i}(t)dt=A_{b}CE_{b}\int_{0}^{t}{\varepsilon_{i}}^{2}(t)dt$$ (15)

$$W_{r}=\int_{0}^{t}A_{b}\sigma_{r}(t)C\varepsilon_{r}(t)dt=A_{b}CE_{b}\int_{0}^{t}{\varepsilon_{r}}^{2}(t)dt$$ (16)

$$W_{t}=\int_{0}^{t}A_{b}\sigma_{t}(t)C\varepsilon_{t}(t)dt=A_{b}CE_{b}\int_{0}^{t}{\varepsilon_{t}}^{2}(t)dt$$ (17)

where *W*_i_(t), *W*_r_(t), and *W*_t_(t) represent the incident, reflected, and transmitted energies, respectively.

Assume that the energy loss at the ends of the specimen and the bar is negligible. According to the law of conservation of energy, the energy dissipation of rock is mainly related to the energy carried by the incident wave, reflected wave, and transmitted wave; the dissipated energy *W*_d_ is expressed as follows:


$$W_{d}=W_{i}-(W_{r}+W_{t})$$ (18)

**Fig 12:**
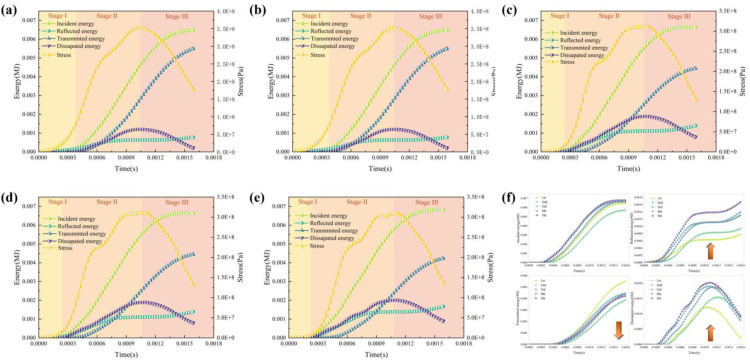
The energy and the stress evolution of the granite specimens of Test 5 with time. (a) 1st impact. (b) 2nd impact. (c) 3rd impact, (d) 4th impact. (e) 5th impact. (f) Energy evolution characteristics.

The energy and the stress evolution of the granite specimens subjected to coupled static–cyclic impact loading with time could be described by three stages (Fig 12a-12e and Table 5). In the stage I, the initial phase is characterized by minimal energy levels and it is characterized by a gradual increase in stress. This stage marks the initiation of mechanical response, with the specimens undergoing the mode transformation of plastic-elastic to plastic with the increase of the number of impacts. In stage II, the rock is in stage of the elastic-plastic deformation and damage growth phase, and the incident energy and stress rise rapidly, while the other energies increase gradually. The incident energy enters a plateau phase very early, and the transmission energy and time curves exhibit a shift to the right as the number of impacts increases, which also indicates an escalation in the damage incurred by the specimen. A salient feature of this phase is the transformation of a considerable portion of the incident energy into damage energy and plastic deformation energy, resulting in a sustained increase in the dissipated energy and a coincident time with a peak in the dissipated energy and the stress. In stage III, the incident energy approaches a constant level, and the stress and the dissipated energy decrease. Subsequently, a portion of this energy is converted into transmitted energy and reflected energy. In addition, the reflected energy is determined by the matching of the wave impedance between the bar and the specimen. The wave impedance remains consistent, and the reflectivity exhibited by the material remains consistent as well. However, the reflected and time curves exhibit a slight upward trend at their terminal point, and a proportion of the energy is converted into transmitted energy and reflected energy, and the proportion of energy used for internal damage and deformation of the rock decreases, which is manifested as a decrease in the dissipated energy and time curve. There is also a reason to believe that a portion of the dissipated energy is the elastic energy stored by the specimen because of the specific rebound characteristics of the stress-strain curve. During the later wave loading, the elastic energy stored within the specimen is released, resulting in a reduction in the dissipated energy and an increase in both the transmitted energy and the reflected energy.

**Table 5:** Energy distribution subjected to coupled static–cyclic impact loading.

Test	Number of cycles	Incident energy (KJ)	Reflected energy (KJ)	Transmitted energy (KJ)	Dissipated energy (KJ)	Peak value of dissipated energy (KJ)
**Test 1**	1	6.05	1.11	3.67	1.28	1.44
	2	5.99	1.03	3.76	1.2	2.01
	3	4.62	0.74	2.98	0.90	1.35
	4	6.54	1.56	4.04	0.93	1.78
**Test 2**	1	6.25	0.56	4.93	0.75	1.47
	2	5.38	1.22	2.97	1.18	1.96
	3	5.9	1.56	3.28	1.06	2.02
**Test 3**	1	5.24	0.81	4.43	0.00031	0.59
	2	5.16	0.77	3.21	1.18	1.53
	3	6.58	1.23	4.23	1.12	1.79
	4	6.98	1.18	4.12	1.68	2.31
	5	4.73	0.97	2.61	1.15	1.62
**Test 4**	1	6.57	0.98	5.12	0.47	1.54
	2	5.51	0.99	3.87	0.65	1.50
**Test 5**	1	6.49	0.78	5.50	0.21	1.21
	2	5.72	0.94	3.88	0.89	1.54
	3	6.64	1.40	4.45	0.80	1.90
	4	6.69	1.31	4.43	0.95	1.88
	5	6.80	1.67	4.23	0.90	2.02

Although the incident energy is not consistent, it is also possible to identify some consistent features (Fig 12f). The reflected energy and time curves shift upward with the increase in the number of impacts. On the contrary, the transmitted energy and time curves shift downward with the increase of the number of impacts. Due to the accumulation of internal damage, the energy is more difficult to transmit and is more reflected. In addition, the dissipated energy and time curves shift upward, even when the incident energy of the second impact is relatively small. It indicates that the energy absorption capacity has been enhanced in the early stage, and the growth of dissipated energy slows down due to damage saturation in the later stage.

**Fig 13:**
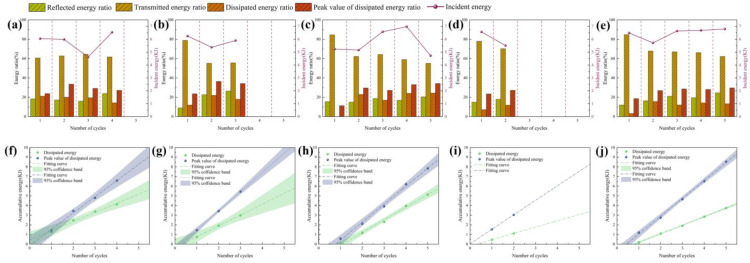
Energy proportion and accumulative dissipated energy. (a) Test 1. (b) Test 2. (c) Test 3. (d) Test 4. (e) Test 5. (f) Test 1. (g) Test 2. (h) Test 3. (i) Test 4. (j) Test 5.

The ratio of other energies to incident energy is presented in the form of a columnar diagram (Fig 13a-13e), it can be observed that the reflected energy, dissipated energy and the peak value of dissipated energy exhibit an increasing trend with the increase of the number of impacts, while the transmitted energy shows a decreasing trend. The cumulative dissipated energy and the peak value of cumulative dissipated energy have a linear relationship with the number of impacts (Fig 13f-13j). With the increase of axial pressure and confining pressure, the cumulative energy of the peak value of dissipated energy exhibits a tendency to increase.

## Failure mode and fracture mechanism

### Failure mode of granite samples subjected to coupled static–cyclic impact loading

**Fig 14:**
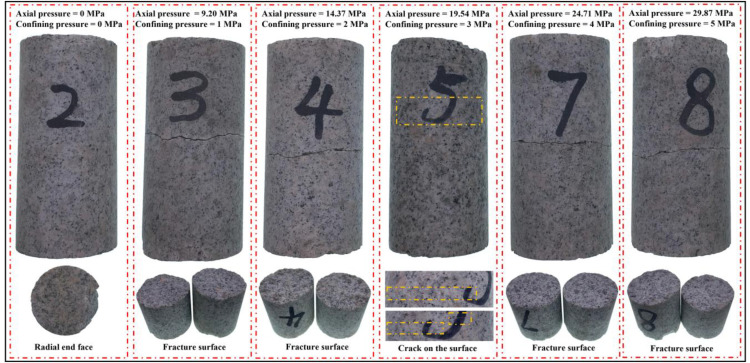
Failure mode subjected to coupled static–cyclic impact loading.

Failure modes of granite samples subjected to coupled static–cyclic impact loading are shown (Fig 14). It can be observed that the failure mode is tensile-dominated failure perpendicular to the axial direction, and there is compression-shear failure at the terminal end of the specimen. Based on the waveform diagram (Fig 9a), the waveforms of the incident wave are symmetrical on both sides of the peak, but the transmitted waves have an obvious difference on both sides of the peak. It is considered that it is the nonlinear effect of wave propagation in rock [24], and Tong et al. [25] have proved the nonlinear dynamic response. In a homogeneous medium, small-amplitude waves propagate at a constant velocity. For finite amplitude waves, velocity varies with local acoustic disturbances, causing waveform distortion as faster waves overtake slower ones, leading to discontinuity over distance [24]. In other words, the incident wavelength is approximately 224 μs, and the transmitted wavelength (about 261 μs) is 37 μs longer than the incident wavelength, and the additional 37 μs duration corresponds to a tensile stress wave. Furthermore, the tail of the transmitted wave also appears as a tensile wave without the specimen (Fig 8c), which is consistent with the phenomenon observed by Tong et al. [25]. The classification of the stress wave as a compression wave or tension wave is determined by the direction of its state change rather than the compression or tension of the medium. The specimen experiences a state of compression under a static load. The incident wave loading consists of a loading phase and an unloading phase. If the stress amplitude in the loading phase is lower than the static preload, the state of the specimen remains unchanged. However, when the stress amplitude in the loading phase exceeds the static load, the specimen enters a state of compression. As the stress amplitude increases, the compression effect intensifies until the specimen reaches its maximum compression displacement. In the unloading phase, the specimen is observed from the point of maximum compressive displacement. Despite being subjected to a compressive wave, the specimen is actually in a tensile state. If the stress wave amplitude in the unloading phase is lower than the static preload, the specimen should remain unchanged under slow loading conditions. Nevertheless, the specimen may undergo a brief oscillation process due to the rapid propagation of the stress wave.

### The transverse relaxation time spectra distribution and pore proportion

Low-field Nuclear Magnetic Resonance (NMR) (MacroMR02-110H-D, China) has been used to test rock samples subjected to coupled static–cyclic impact loading. The magnet type is a permanent magnet, the magnetic field intensity is 0.3 ± 0.05 T. The maximum limited detection dimensions are a diameter of 150 mm and a length of 100 mm. The magnetic field stability is less than300 Hz/Hour, and the magnet temperature is precisely controlled by nonlinear constant temperature and can be adjusted within the range of 25–35 °C. The pulse frequency range is 1–30 MHz, the frequency control accuracy is 0.1 Hz, the pulse accuracy is 100 ns, the radio frequency (RF) transmission power is a peak output greater than 300 W, and the maximum sampling bandwidth is 2000 kHz. The maximum number of echoes of the Carr Purcell Meiboom Gill (CPMG) is 18000, the sampling rate is 50 MHz, and the frequency resolution is 0.0000007 Hz. The rock is first subjected to vacuuming through a water saturation device, then the pressure is applied. After 8 hours, the sample is taken out. A thin layer of cling film is wrapped around the surface of the sample to prevent water evaporation.

The mesoscopic structure inside rocks is characterized by detecting the energy state of hydrogen protons in water. Hydrogen nuclei possess magnetic moments that align with an external magnetic field, inducing equilibrium and enabling energy absorption. radio frequency pulses cause precession, and upon pulse removal, magnetic moments re-align, emitting detectable signals. Relaxation times, influenced by fluid-pore interactions and surface relaxivity, exhibit distinct types in fluid-filled rock pores, with surface relaxation dominant at solid-water interfaces [26]. The transverse relaxation time (T_2_) of the material is predominantly governed by the surface relaxation time, which, in turn, is contingent upon the surface relaxation rate and the specific surface area of the pores, as delineated in Eq. (19) [27].


$$T_{2}\approx T_{S}=\rho_{2}{\left(\frac{S}{V}\right)}_{pore}$$ (19)

$$\rho_{2}{\left(\frac{S}{V}\right)}_{pore}=F_{S}\left(\frac{\rho_{2}}{r}\right)$$ (20)

where *ρ*_2_ is the surface relaxation rate (*ρ*_2_ is 10 μm/s in our experiments), *S* and *V* are the surface area and volume of the pore, *F*_s_ is the pore shape factor (the values of spherical pore and columnar pore are 3 and 2, respectively), and *r* is the pore size.

**Fig 15:**
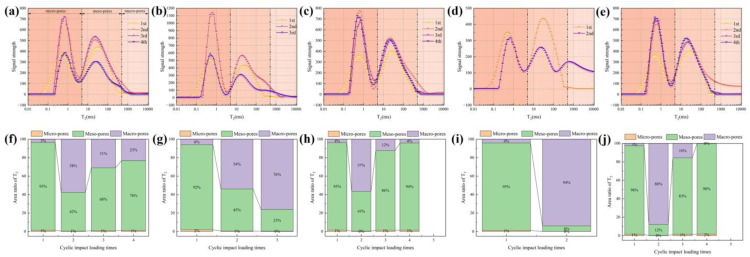
The T_2_ spectra distribution and pore proportion. (a) Test 1. (b) Test 2. (c) Test 3. (d) Test 4. (e) Test 5. (f) Test 1. (g) Test 2. (h) Test 3. (i) Test 4. (j) Test 5.

The NMR detection was performed on unimpacted and impacted samples, while the fragments of the samples were not tested (Table 6). The T2 spectra distribution and pore proportion are shown (Fig 15). The pore is divided into three intervals: micro-pores (r < 0.1 μm), meso-pores (0.1 μm ≤ r < 10 μm), and macro-pores (r ≥ 10 μm) [27]. Relaxation time reflects the movement characteristics of fluid molecules in pores. The longer the relaxation time, the larger the pore size. The higher the signal intensity peak height, the more pores in this aperture range. The number of meso-pores is generally more than that of micro-pores in the original rock samples, i.e., meso-pores play a leading role in rock fracture (the 1st describes the un-impact specimen). After the first impact loading, the T_2_ curve shifts to the right and the signal intensity peak height increases, but the pore proportion is basically unchanged, indicating that the size of micro-pores increases. Meanwhile, the meso-pores transform to the macro-pores, it manifests as a decrease in the proportion of meso-pores and an increase in the proportion of macro-pores. With the increase of impact times, the size of meso-pores and macro-pores increases. The proportion of meso-pores and macro-pores is complementary, and macro-pores increased first and then decreased, or increased all the time. In addition, there are cases where the signal intensity peak height of T2 spectra of micro-pores is basically the same as that of the original sample(Fig 15a-15b and Fig 15d), and it may provide some justification for tensile fracture.

**Table 6:** The Cyclic impact loading times compared NMR detection times.

	Test 1	Test 2	Test 3	Test 4	Test 5
**Cyclic impact loading times**	4	3	5	2	5
**NMR detection times**	4	3	4	2	4

### Fracture surface roughness and fracture surface morphology of samples

**Fig 16:**
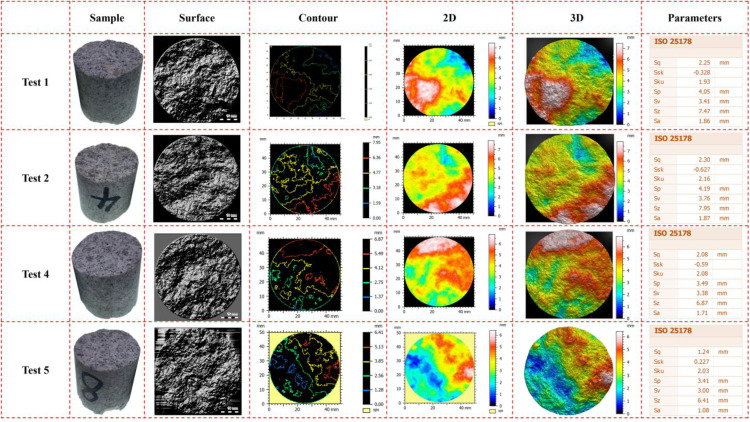
Fracture surface morphology of samples.

Fracture surface roughness is a key parameter for characterizing post-fracture material morphology. The PS50 compact optical profilometer (NANOVEA, American) could be used for measuring the fracture surface roughness [17]. The test outcomes regarding the surface topography and roughness parameters of rock samples of Test 1, Test 2, Test 4, and Test 5 are depicted (Fig 16). The “Surface” image showcases the microscopic texture of each surface of the samples, thereby reflecting the actual roughness. The “Contour” representation presents the surface height variations in the form of contour lines, which is conducive to comprehending the undulating features. The “2D” image is a pseudo-color plan of surface height, while the “3D” image represents the three-dimensional topography map, both of which provide an intuitive visualization of the three-dimensional fluctuations of the surfaces. Following the ISO 25178 [28] standard, roughness parameters, including Root Mean Square Height (Sq), Skewness (Ssk), Steepness (Sku), Maximum Peak Height (Sp), Maximum Valley Depth (Sv), Maximum Height (Sz), and Arithmetic Mean Height (Sa), were tabulated. These parameters were employed to quantitatively characterize the surface roughness features, enabling a more precise and systematic analysis of the surface roughness properties of rock samples.

The surface textures of Test 1, Test 2, and Test 4 can be generally characterized as rough. These surfaces exhibit drastic gray level variations, accompanied by distinct ravines and bumps in their texture patterns. In contrast, the surface texture of Test 5 is relatively delicate, with a more uniform grayscale distribution. This uniformity indicates that the microscopic fluctuations on the rock surface of Test 5 are significantly smaller, while the physical roughness of the surfaces in the first three test groups is notably higher. For instance, the texture of Test 1 features a pronounced contrast between dark sagging areas and light protruding bumps, whereas the texture depth transition in Test 5 is much gentler. Regarding Test 2, the gray level distribution and texture morphology of its surface texture are relatively disordered, with substantial variations in texture characteristics across different regions. Although the texture of Test 4 is rough, the variations in texture density and depth in local areas display a certain degree of repeatability. This suggests that the surface texture of Test 4 possesses slightly stronger periodicity or regularity compared to that of Test 2.

The contour shape of Test 2 is more pronounced irregular distortion, with a substantial disparity in the contour direction between the center and the edge. Although the contour lines of Test 1 also exhibit irregularity, they predominantly approximate a quasi-circular form. This characteristic suggests that the surface height distribution of Test 1 possesses slightly better symmetry compared to that of Test 2. The contour lines of Test 4 are the densest among the samples. Within the same horizontal distance, these contour lines span a greater number of elevation levels, indicating a steep variation in surface height. Conversely, the contour lines of Test 5 are the sparsest, reflecting gentle height changes across the surface.

The distinct concentrated warm-color regions (high-elevation areas) and the concentrated cool-color regions (low-elevation areas) are observed in Test 1, with a clear spatial separation between the high and low areas. When examining the 3D morphology of Test 2 from multiple perspectives, significant morphological differences are evident in local bulges and depressions; for instance, a peak on one side coexists with a wide valley on the other. The Test 4 exhibits an extremely rapid color transition from cool colors to warm colors, with a large color span within small regions, which indicates substantial local height differences. In contrast, Test 5 shows a slow color gradient, with minimal color variation across the same area, reflecting a relatively uniform surface height distribution. In terms of 3D topography, Test 4 presents a steep peak and deep valley configuration, displaying the most pronounced three-dimensional fluctuations. Conversely, the 3D morphology of Test 5 resembles a smooth hill, characterized by very slight undulations. These observations further validate the earlier findings regarding surface roughness and texture, collectively providing a comprehensive characterization of the topographical variations across the test groups.

**Fig 17:**
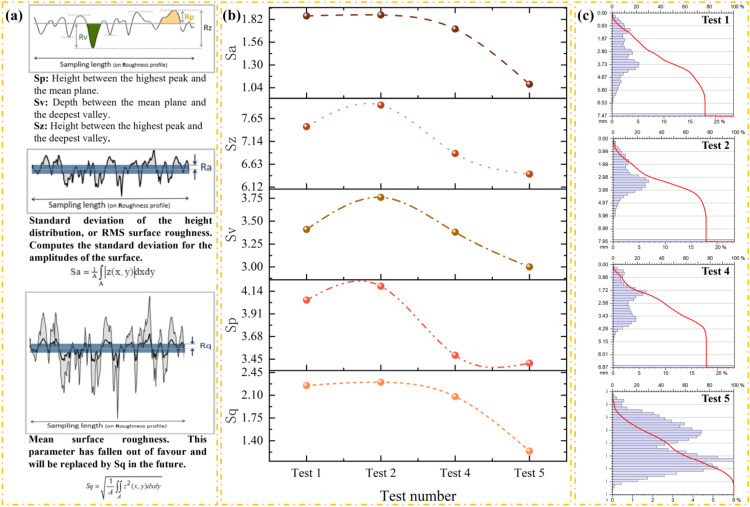
Fracture surface roughness. (a) Meaning of parameters. (b) The variation characteristics of fracture surface roughness. (c) Depth histogram and Abbott-Firestone curve.

The variation characteristics of the influence of experimental conditions on the fracture surface roughness are depicted (Fig 17), and it describes the calculation method of the fracture surface roughness (Fig 17a). As the axial pressure and confining pressure increase incrementally, a discernible trend emerges wherein the roughness parameters exhibit a gradual decline. This downward trend in roughness parameters strongly suggests that the fracture surfaces of the rock samples become progressively smoother (Fig 17b). Such an observation implies that higher levels of axial and confining pressures significantly influence the formation and evolution of fracture surfaces, altering their topographical characteristics and reducing surface irregularities. It describes the Depth histogram and the Abbott-Firestone curve (Fig 17c). The Depth histogram allows for observing the density of the distribution of the data points in the surface being studied. The vertical axis is graduated in depths, and the horizontal axis is graduated in % of the whole population. It tells how many data points lie at a particular depth (or height). The depth distribution graph is used to compute the Abbott-Firestone curve. This curve is also called the Bearing area curve or Material ratio curve. The Abbott Firestone curve is the cumulative function of the depth distribution function. The horizontal axis represents the bearing ratio (in %) and the vertical axis the depths (in the measurement unit). Test 1 exhibits concentrated regions of bulges and depressions (Fig 16), which is reflected in the 2D pseudo-color map as large color blocks. And its Depth histogram shows an obvious long tail on the right (Fig 17c). Such a long tail in the height distribution is attributed to the presence of local extreme heights on the surface, such as sharp peaks and deep valleys. It is indicated that although the proportion of such areas is small, the statistical impact on the overall height is significant. Conversely, Test 5 features the finest surface texture, characterized by shallow textural details in the surface graph and a gentle 3D morphology (Fig 16). And its Depth histogram presents a sharp peak with a narrow span, and the Abbott-Firestone curve shows a steep rise. These characteristics imply that the more uniform the surface, the more concentrated the height distribution.

### Characteristics of velocity field of granite samples based on numerical simulation

Numerical simulation serves as a valuable complement to experimental observations by capturing phenomena that are inaccessible through experiment. It is particularly beneficial for analyzing the rock dynamic fracture mechanism, as it can unravel complex processes such as the initiation and propagation of microcracks, the evolution of stress fields, and the fracture behavior. By bridging these observational gaps, numerical simulation enhances the comprehensiveness of mechanistic interpretations.

**Fig 18:**
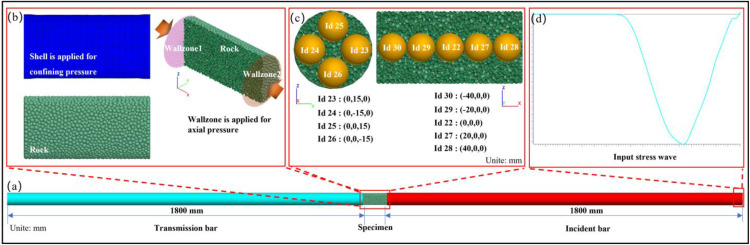
Numerical model of SHPB. (a) FE-DEM of SHPB. (b) The coupling of FLAC and PFC. (c) The measure spheres. (d) The loading wave.

A numerical simulation model for SHPB has been established utilizing the coupled PFC-FLAC method (PFC: Particle Flow Code; FLAC: Fast Lagrangian Analysis of Continua), and the axial pressure is applied by utilizing the generated wall through the zone, while the confining pressure is exerted via the application of a shell. Both pressures are applied in a servo-controlled manner to ensure that the preset axial and confining pressures for the particles are accurately achieved (Fig 18). To ensure the model accurately reflects real-world behavior, the microscopic rock parameters were calibrated through extensive trial-and-error tests, to match the macroscopic mechanical responses. The calibration results, along with the microscale properties of the specimen, are summarized (Table 7) [17].

**Table 7:** The micro-scale properties of rock.

Minimum particle radius (mm)	*R*_min_	1.2
**Ratio of particle radius**	*R*_min_/*R*_max_	1.5
**Density (kg/m**^**3**^)	*ρ*	2707
**Damping coeﬃcient**	*η*	0.05
**Normal to shear stiﬀness ratio**	*k*_*n*_/*k*_*s*_	2.28
**Bond effective modulus**	E*	7.69
**Eﬀective modulus (GPa)**	*E*	39.31
**Friction coeﬃcient of particle**	*μ*	0.5
**bond tensile strength (MPa)**	*σ*_*c*_	35
**bond cohesion strength (MPa)**	*c*	80
**bond friction angle**	*φ*_*b*_	20°

**Fig 19:**
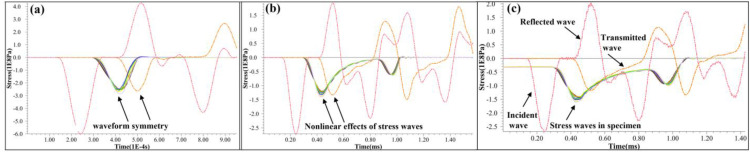
Characteristics of stress wave propagation. (a) High strain rate without axial pressure and confining pressure. (b) Low strain rate without axial pressure and confining pressure. (c) Applied axial pressure and confining pressure.

The waveform characteristics of stress wave propagation under diverse loading conditions, including variations in strain rate, the presence or absence of axial compression, and confining pressure, are presented (Fig 19). Under high-strain-rate impact conditions in the absence of axial and confining pressures, the rock sample undergoes fracture (Fig 19a). Notably, the transmitted waves and internal stress waves of the sample display symmetrical characteristics. The undamaged rock samples display notable nonlinear characteristics in transmitted waves and internal stress waves of the sample (Fig 17b-19c) [29]. The finding aligns seamlessly with the experimental the wavelength of the transmitted wave exceeds that of the incident wave. Through meticulous frequency spectrum analysis, Tong et al. [25] demonstrated that stress waves undergo significant nonlinear transformations upon traversing rock specimens. Specifically, they identified the additional wavelength component of the transmitted wave, relative to the incident wave, as a tensile wave. It is also indicated that a significant plastic deformation occurred after impact (Fig 11).

**Fig 20:**
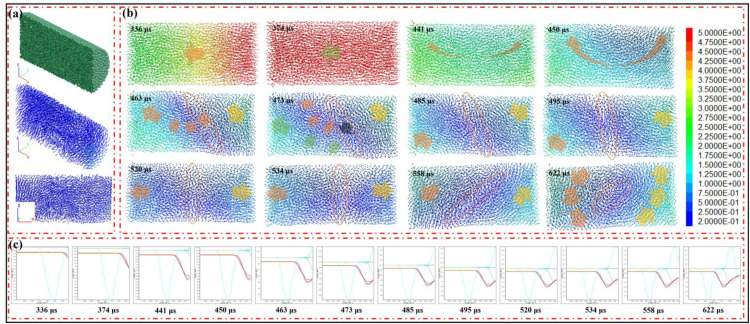
Characteristics of the velocity field. (a) Slice perspective. (b) Velocity field. (c) Stress wave propagation process.

The sample is axially sliced, and the particles are then visualized as arrows, where the arrow directions represent the velocity directions, and colors denote the magnitudes of the velocities. The visualization method enables the observation of the variation characteristics of the particle velocity field, providing insights into the dynamic motion behavior of particles within the sample under the given experimental or simulated conditions (Fig 20a). It can be seen that, before the monitored stress within the specimen attains its peak value, the particle velocity vector exhibits a strict collinear alignment with the principal axis, congruent with the propagation direction of the stress wave (Fig 20b-20c). Following the stress peak, the propagation direction of the velocity field starts to deviate, as evident from the images captured at 441 μs and 450 μs. Subsequently, at 463 μs, a transition region emerges within the velocity field. The velocity fields on either side of this transition region exhibit a near-symmetrical configuration, with velocity vectors oriented in opposite directions. As time elapses, the transition zone gradually rotates, shifting from an initial inclination towards the direction of stress wave propagation to a perpendicular orientation relative to it. Notably, at this juncture, the wavelength of the monitored stress wave within the sample aligns closely with that of the incident wave. It is distinct that the pattern observed in the velocity field serves as a strong indicator of the presence of tensile stress waves. As the stress wave continues to propagate, the transition region further tilts, orienting itself towards the incident bar.

## Discussion

### The schematic diagram of rock fracture based on NMR

**Fig 21:**
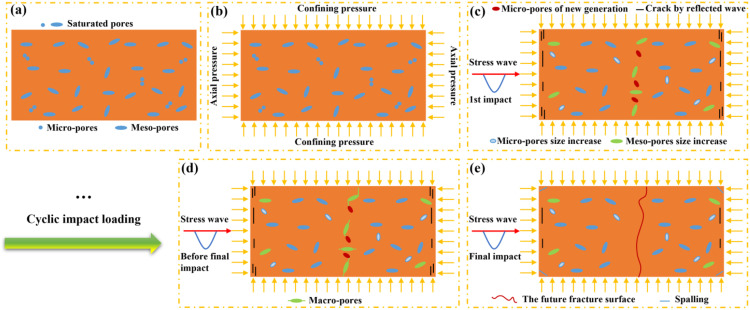
The schematic diagram of rock fracture based on NMR.

The 1st curve represents the T2 spectrum of pores in initial granite samples (Fig 15a-15e). The relaxation time in the initial state of the samples is less than 0.1ms (Fig 21a). Upon experiencing preloading and loading, the component of relaxation time at 0.1ms disappears, indicating a rightward shift in the curve. Concurrently, the peak value of the T2 spectrum in the micro-pores region increases, suggesting the interconnection of smaller micro-pores or an enlargement in pore size [30], as depicted in the schematic diagrams (Fig 21b-21c). The peak value of the T2 spectrum in the meso-pores also exhibits an increase. However, before the final impact, the peak value of the T2 spectrum of micro-pores in the sample returns to the level of the initial peak, indicating that cyclic impacts generate new small pores due to the action of stress waves. These newly formed pores contribute to macro-pores, crack propagation, and the formation of fracture surfaces. The peak value of the T2 spectrum in the meso-pores decreases and falls below the initial state, yet there is an indication of a third peak of the T2 spectrum in the meso-pores, and it is indicated that the diameter of the meso-pores is expanding [31]. Sometimes, there may also be cases where meso-pores develop into macro-pores. Meso-pores play a pivotal role in macroscopic crack propagation (Fig 21d-21e).

### The fracture mechanism based on numerical simulation

The velocity vector directions on both sides of the specimen are opposite to the vicinity of the specimen center as the boundary within 200 μs. According to the formula for stress calculation, the specimen undergoes a shearing and tensing process. The velocity vectors are along the axial direction and in opposite directions for 16 μs, and the specimen undergoes a stretching process.


$$\sigma_{particle}=\rho_{sample}v_{particle}C_{sample}$$ (21)

Where *σ*_*particle*_ is the stress of the particle, *ρ is* the density of the sample, *v*_*bul*_ is the velocity of the particle, and *C* is the velocity of the longitudinal wave.

**Fig 22:**
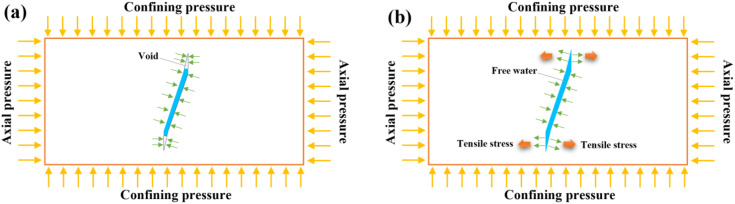
Fracture induced by water and tensile stress.

Jin et al. [32] found that the axial stress has a significant impact on the propagation of stress waves. When there is no axial stress, there is almost no tensile wave. However, when the specimen has axial stress, a tensile wave appears at the tail of the stress wave. The greater the axial pressure, the larger the tensile wave at the tail of the stress wave. And it is consistent with the phenomenon observed by Tong et al. [25]. Du et al. [33] found that the free water fails to get to the newly formed cracks during dynamic loading (Fig 22a); the free water in the specimen can diffuse to the newly formed crack tips after each impact (Fig 22b). The stress wave enhances the role of free water at the crack tip (Fig 22b). Therefore, the crack propagation is longer in the middle part of the specimen compared to other locations, and the fracture surface is more likely to occur in the middle part of the specimen.

### Horizontal discontinuity by the intersection of shear planes

**Fig 23:**
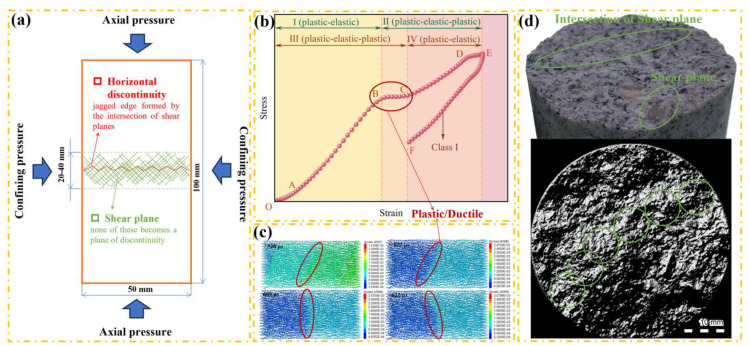
Horizontal discontinuity by the intersection of shear planes. (a) Santarelli et al. (1989). (b) Plastic stage in the stress-strain curve. (c) Displacement field based on numerical simulation. (d) Intersection of shear planes observed on the fracture surface.

Santarelli et al. [34] found that the specimen exhibits ductile behavior at high confining pressures. A large number of shear planes are developed in a horizontal band 2–4 cm wide, but none of these becomes a plane of discontinuity. The only discontinuity in the sample is horizontal with a jagged edge formed by the intersection of various shear planes having different orientations (Fig 23a). Similarly, the intersection of shear planes is observed on the fracture surface (Fig 23d).

By observing the displacement field during the specimen loading process (Fig 23c), only displacement information at partial time points is presented. In the displacement field, two zones appear in the middle of the specimen from 533 μs to 682 μs. The displacement value in the zone close to the incident bar is relatively large, while that in the zone close to the transmission bar is relatively small. The displacement within each of the two zones is uniform, which means the strain is also uniform. A displacement gradient occurs at the junction of the two zones, indicating that plastic deformation or crack propagation exists at the junction [35]. A plastic stage [36] is also observed in the stress-strain curve (Fig 23b). Meanwhile, the nonlinear characteristics of the transmitted wave also provide evidence for this phenomenon.

### Deformation of the specimen within the compression device

**Fig 24:**
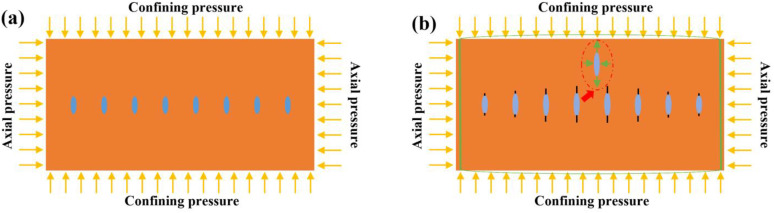
The schematic diagram of deformation of the specimen within the compression device.

The specimen is placed in a confining pressure device and wrapped in a specially-made rubber sleeve. Confining pressure is applied to the rubber sleeve through hydraulic oil and then transmitted to the specimen. Axial pressure is applied to the rigid bar via hydraulic oil and subsequently transferred to the specimen. Compared with the rigid bar, rubber is a flexible material and can undergo slight deformation. The experimental operation procedure is to apply the confining pressure first. At this time, the confining pressure acts on both the rubber and the specimen, and their deformations under the action of force are consistent. After the axial pressure is applied, since the axial pressure is greater than the confining pressure, the specimen tends to expand radially, but this expansion is restrained by the confining pressure. The flexibility of the rubber may cause the middle part of the specimen to slightly bulge due to insufficient constraint, which in turn leads to the concentration of bending stress and promotes the initiation of cracks in the middle part. Meanwhile, this bending phenomenon depends on the thickness and hardness of the rubber (Fig 24). In conclusion, this is a speculation about the experimental system. It lacks data support and may be one of the factors causing the fracture of the middle part of the specimen.

## Conclusions

This study systematically investigates the radial fracture mechanism of saturated granite under coupled static–cyclic impact loading through experimental tests, stress wave analysis, numerical simulations, and microscopic characterization. The main conclusions are as follows.

(1) Under coupled static–cyclic impact loading, saturated granite primarily exhibits radial tensile-dominated failure, with compression-shear failure localized at the specimen ends. This failure pattern is closely related to the combined effects of static preload (axial and confining pressures) and cyclic impact-induced stress waves, corresponding to engineering depths of 156.20–582.07 m.
(2) Stress waves propagate nonlinearly in the granite specimens. The transmitted wave wavelength is longer than that of the incident wave, with the additional 37 μs attributed to tensile stress waves. This phenomenon is confirmed by waveform analysis, which reveals that the tensile stress is one of the factors causing radial tensile failure.
(3) Numerical simulations using the coupled PFC-FLAC method demonstrate that the particle velocity field in the specimen undergoes a symmetrical transition. Velocity vectors on either side of a central transition zone orient in opposite directions, directly reflecting the action of tensile stress. Meanwhile, the free water in crack tips assists in enhancing the crack propagation.
(4) Two distinct zones appear in the middle of the specimen in the displacement field, corresponding to the plastic deformation stage in the stress-strain curve, further verifying the plastic damage process during fracture.
(5) NMR analysis shows that before the final impact, the peak of the T₂ spectrum of micro-pores approximately returns to its initial state.
(6) Energy evolution characteristics indicated that with increasing impact cycles, dissipated energy and reflected energy increased, while transmitted energy decreased; cumulative dissipated energy showed a linear correlation with the number of impacts.
(7) Fracture surface roughness parameters decrease with increasing axial and confining pressures, indicating that higher static pressures lead to smoother fracture surfaces. This trend is attributed to the inhibition of irregular crack propagation by elevated confining pressure and the more uniform energy dissipation under higher axial pressure.
